# Melanoma/CSPG4-Enhanced Collagen-Mediated Contact Guidance Requires Mutant Active BRAF and the CSPG4 Core Protein Cytoplasmic Domain

**DOI:** 10.1007/s12195-025-00882-x

**Published:** 2025-12-23

**Authors:** G. Thrivikraman, J. Yang, M. Price, A. Giubellino, J. B. McCarthy, R. T. Tranquillo

**Affiliations:** 1 Department of Biomedical Engineering, University of Minnesota, Minneapolis, MN 55455 USA; 2 Department of Laboratory Medicine and Pathology, University of Minnesota, Minneapolis, MN 55455 USA; 3 Masonic Cancer Center, University of Minnesota, Minneapolis, MN 55455 USA; 4 Department of Chemical Engineering & Materials Science, University of Minnesota, Minneapolis, MN 55455 USA; 5 Present Address: Department of Biotechnology, Bhupat & Jyoti Mehta School of Biosciences, Indian Institute of Technology Madras, Chennai, 600036 India

**Keywords:** Contact guidance, Melanoma, Aligned collagen, Chondroitin sulfate proteoglycan-4

## Abstract

**Introduction:**

Chondroitin sulfate proteoglycan-4 (CSPG4) is a transmembrane cell surface proteoglycan that promotes malignant progression in melanoma. Elevated CSPG4 expression in melanoma cells is associated with several malignant phenotypic properties, including increased tumor cell invasion, tumorigenic potential, and metastasis.

**Methods:**

Magnetically aligned collagen gels with entrapped cells were used to model the aligned extracellular matrix in the tumor microenvironment and to identify the key role of CSPG4 in sensing contact guidance.

**Results:**

The data show that CSPG4-expressing WM1552C Radial Growth Phase (RGP) melanoma cells exhibit enhanced contact guidance along with increased migration speed in contrast to paired counterparts that lack CSPG4. This required the presence of a pERK 1,2 phospho-acceptor site on the cytoplasmic tail of the core protein. Furthermore, short-term treatment of CSPG4-expressing cells with the clinically used mutant active BRAF inhibitor vemurafenib reduced both guidance and speed.

**Conclusions:**

These findings support the role of CSPG4 overexpression and mutant active BRAF-in promoting increased contact guidance. The results are discussed in terms of expanding what is known about the potential tumor biology and clinical implications of CSPG4-related impact on malignant invasion during early phases of melanoma progression.

**Supplementary Information:**

The online version contains supplementary material available at 10.1007/s12195-025-00882-x.

## Introduction

Chondroitin sulfate proteoglycan 4 (CSPG4) is a cell surface oncoantigen associated with malignant progression in melanoma [1, 2]. CSPG4 functions as a cell surface ‘sensor’ of the remodeling tumor microenvironment to promote activation of multiple oncogenic pathways in tumor cells [1]. These pathways are associated with increased tumor cell growth, motility, invasion, and survival [2]. Structurally, CSPG4 (and the rat homologue NG2) is expressed as a type 1 transmembrane glycoprotein, a portion of which is decorated with chondroitin sulfate glycosaminoglycan. The core protein consists of a large extracellular region, a transmembrane region, and a relatively short cytoplasmic domain. CSPG4 functions by multiple mechanisms to enhance oncogenic signaling pathways and a mesenchymal transition transcriptome. These include enhancing growth factor presentation to their cognate membrane-associated tyrosine kinase receptors, functionally modifying key cell adhesion-promoting pathways, and stimulating tumor cell growth/motility-promoting pathways [1, 2].

In melanomas, dysregulation of the MAPK pathway occurs frequently due to activating mutations in the BRAF (and RAS) genes or other genetic or epigenetic modifications, leading to increased oncogenic signaling activation, which promotes tumor cell proliferation, invasion, metastasis, migration, survival, and angiogenesis [3, 4]. BRAF is a serine-threonine kinase that serves as an effector of RAS in the mitogen-activated protein kinase (MAPK or ERK 1,2 ) signaling pathway [4]. BRAF mutation V600E (and related mutations in this codon) yield constitutive activation of the MAPK/ERK pathway via RAS/RAF/MEK/ERK 1,2 signaling in melanoma [5]. Studies show that the CSPG4 expression in melanoma tumor cells has been functionally linked to enhancing the activity of mutant BRAF [6, 7]. V600E and V600K activating mutations exhibit a prevalence of ~ 40% and ~ 5% in patients, respectively [8]. In particular, melanoma cells harboring the BRAF V600E mutation exhibit increased invasion, which is facilitated by promoting an invasive transcriptome [9]. Pharmacological inhibitors, such as Vemurafenib, which selectively bind to the ATP-binding site of BRAF V600E kinase and inhibit its activity, result in potent inhibition of ERK 1,2 phosphorylation and serve to limit tumor cell growth [10]. In animal model experiments, vemurafenib limits growth and yields regression of tumors possessing the BRAF V600E mutation. Furthermore, in clinical trials of melanoma patients expressing the BRAF V600E mutation, as well as in phase I, II, and III trials of patients with metastatic melanoma, vemurafenib therapy produces a high initial response rate, although development of long-term resistance remains a challenge to its efficacy [10].

Increased expression of CSPG4 is observed throughout melanoma progression, including early in radial growth phase (RGP), later in vertical growth phase (VGP) tumors, as well as in metastases [11]. In RGP melanoma-derived tumor cells, CSPG4 expression enhances both anchorage-independent growth *in vitro* and tumor formation in a xenograft injection model [7], implicating CSPG4 in promoting early phases of tumor growth and progression. The invasion of RGP cells from primary tumors involves increased migration into a collagen-rich dermal microenvironment, leading to the formation of VGP tumors, which intravasate into blood vessels or lymphatics and form metastasis to distant organs [4, 12].

Tumor cell migration and invasion from the primary tumor involve complex mechanosensitive mechanisms, where the organization, density, and stiffness of collagen in the fibrotic stroma play significant roles [12, 13]. Notably, the structural orientation of tumor-associated fibrotic collagen can enhance contact guidance, the phenomenon where cells orient and migrate bi-directionally in response to anisotropic topographical features, such as parallel grooves on a 2D substrate or aligned fibrils in 3D collagen, and even aligned fibers in tissue [13–15]. Increased tumor migration is often correlated with the presence of bundles of linear, aligned collagen fibers oriented perpendicularly to the tumor boundary, thereby serving as a prognostic marker to help predict clinical outcomes for invasive carcinoma [16, 17]. Consistent with this, the role of contact guidance along aligned collagen fibers radiating outward from the tumor in promoting tumor cell metastasis was first demonstrated in live breast tumors *ex vivo* [18] and later in B16F10 melanoma invasion *in vivo* [19]. Furthermore, *in vitro* studies revealed that invasive breast cancer cell lines exhibit different migration behaviors (mesenchymal MDA-MB-231 vs. amoeboidal MTLn3) and show varying degrees of sensitivity to contact guidance in response to collagen fibrils aligned on a planar substrate, indicating distinct guidance sensing mechanisms based on migration modes [20, 21]. Additionally, creating wavy fibril bundles that mimic healthy stroma has been shown to reduce contact-guidance-mediated migration and dissemination, in contrast to linear fiber bundles characteristic of metastasis-prone stroma [22]. This effect can be overridden by the upregulation of actomyosin contractility [22]. Collectively, these studies utilizing 3D aligned collagen models that emulate fiber alignment in the tumor microenvironment provide valuable insights into the biological significance of contact guidance in driving the metastatic cascade. Here, we use magnetically-aligned collagen gels [23] to investigate the signal transduction pathway of melanoma cells entrapped within aligned collagen fibrils.

Previous studies have shown that RGP melanoma cells expressing CSPG4 exhibit increased migration relative to CSPG4 null cells in a scratch wound migration assay over an isotropic fibronectin surface [7]. However, a link between CSPG4 expression in melanoma cells and contact guidance in response to aligned collagen fibrils, has not been previously established. This study demonstrates that CSPG4 expression increases the contact guidance sensitivity of poorly metastatic RGP melanoma cells migrating within aligned collagen fibrils. Furthermore, both contact guidance and migration speed were related to structural features of CSPG4 that impact the ERK 1,2 pathway via alterations in mutant active BRAF. The results demonstrate that CSPG4 expression in RGP melanoma cells with activating BRAF mutations may function to promote migration and enhance the invasion of cutaneous melanomas into the collagen-rich tumor-associated dermis.

## Methods

### Cell Culture

Radial growth phase human melanoma cancer cell line, WM1552C, were cultured in melanoma culture medium constituting 4:1 ratio of MCDB 153 medium (Sigma) with 1.5 g/L sodium bicarbonate (Sigma) and Leibovitz’s L-15 medium (Sigma) with 2 mM L-glutamine (Gibco), supplemented with 5 μg/ml insulin (Sigma), 0.25 mg/mL G418 (Gibco), 2% fetal bovine serum (FBS; Hyclone) and 1% penicillin/streptomycin (P/S; Gibco). All cultures were maintained at 37 °C in a humidified atmosphere containing 5% CO2. For pharmacological inhibition studies, the cell monolayer in the culture flasks was treated with 1 µM Vemurafenib (Stem cell Technology) in melanoma cell culture medium overnight before encapsulation within collagen hydrogels.

### Plasmid Transfection and Generation of Mutant Strains

**CSPG4 construct preparation and generation of stable cell lines:** The full-length CSPG4 construct (CSPG4^WT^) and mutants CSPG4^ΔCD^, and CSPG4^ΔPDZ^, were generated as described previously [7, 24]. Previous studies have also implicated phosphoacceptor sites for PKC and pERK in the cytoplasmic tail of the NG2 core protein in stimulating tumor cell motility and growth [25]. Thus, for the current studies we also prepared CSPG4 mutants in which PKC and ERK phospho threonine residues were replaced with valine (CSPG4^T2252V^ and CSPG4^T2310V^ respectively). They were generated by PCR-site-directed mutagenesis of the wild type CSPG4 construct (primers: T2252V: 5’TCCGAAAACGCAACAAGGTGGGCAAGCATGACGTCC 3’ and 5’GGACGTCATGCTTGCCCACCTTGTTGCGTTTTCGGA 3’; T2310V: 5’TGCAGTTCTGCCGGGTACCCAACCCTGCCC 3’ and 5’GGGCAGGGTTGGGTACCCGGCAGAACTGCA 3’) modifying threonine 2252 or threonine 2310 to valine, respectively. Mutagenesis was carried out using the QuikChange™ II Site-Directed Mutagenesis Kit (Agilent) using the manufacturer’s suggested protocol. All DNA constructs were verified by sequencing at the Biomedical Genomics Center at the University of Minnesota. Stable cell lines expressing each CSPG4 variant construct were generated as previously described [24] and maintained in normal culture media supplemented with 0.25 mg/ml G418 (Gibco).

### Collagen Gel Preparation

Collagen gel solution was prepared based on the protocol described previously [23]. Briefly, acid-solubilized rat tail Type I collagen (3 mg/mL; Gibco) was diluted in a neutralizing solution containing 1 M HEPES (Corning), 1 N NaOH (Sigma), 7.5 % Sodium bicarbonate solution (Sigma), 10x minimum essential medium (MEM) (Gibco), 10% FBS, 1% P/S, 2 mM L-glutamine (Gibco) and 1X melanoma culture medium at volumes required to obtain a final concentration of collagen at 2 mg/mL. For experiments involving cell encapsulation, the confluent cell monolayer was detached from the culture flask by enzymatic treatment with 0.05% trypsin-EDTA. The resulting cell pellet was re-suspended in melanoma culture medium and mixed with the pre-gel mixture to obtain a final cell concentration of 50,000 cells per ml. All the solutions were kept chilled to prevent fibrillogenesis of collagen prior to magnetic field exposure.

### Guidance Chamber and Magnetic Alignment

Contact guidance experiments were carried out as previously described [23]. The guidance chambers used in this study comprised of a central U-shaped acrylic plate sandwiched between two glass slides sealed tightly using a non-toxic silicone adhesive. The central rectangular compartment thus formed had a length of 4 cm, width of 1 cm, and height of 3 mm. Prior to adding gel, the chambers were sterilized using 70% ethanol and thoroughly rinsed in 1X PBS overnight. After loading the collagen gel solution into the middle compartment, the open side of the chamber was firmly sealed using a T-shaped acrylic cap and secured using parafilm to prevent culture media evaporation, as well as to reduce contamination. The guidance chambers were immediately transferred to the bore of an external magnet with a field strength of 9.4 T for at least 1 hr at RT to induce self-assembly and fiber alignment. Confocal reflectance imaging (Nikon A1RMP multi-photon upright microscopy system) was performed to visualize the collagen alignment using a 40X objective lens (CFI Apochromat NIR (Nikon), Numerical Aperture: 0.8). Z-stacks were acquired in Galvano scanner mode with a slice separation of 1 µm, a field of view of 1024 × 1024 pixels, and a lateral resolution of 0.12 µm per pixel. Further, polarimetric imaging was done as previously described [26, 27], to determine the extent of fibril alignment based on the linear birefringence exhibited by the anisotropic fibrillar structures. Briefly, images of collagen gels were captured using an in-house built macroscope equipped with a rotating polarizer and a circular analyzer in transmission mode, taking measurements at various polarizing angles ranging from 0 to 180 degrees. The intensity of transmitted light through the aligned gels at each pixel undergoes sinusoidal oscillation, and the amplitude and phase shift of this oscillation are related to the local alignment direction and strength (retardation), respectively, computed using Mueller matrix analysis of the optical train.

### Time Lapse Imaging & Analysis

After the magnetic alignment of cell-encapsulated collagen gels, the guidance chambers were mounted on an on-stage incubator attached to a motorized X-Y stage of EVOS^TM^ FL Auto Imaging microscope. Subsequently, time-lapse images were captured for a period of 6-8 h over an interval of 20 min. An objective of 20X magnification was used to capture bright field images and Z-stack images were acquired in a 10 µm range above and below the focal plane with a step resolution of 1 µm. The resulting maximum intensity projection image sequence was then imported into ImageJ software, and the cell displacement trajectory was traced using the ‘Manual Tracking’ plugin. The obtained x-y co-ordinates of the projected 3D cell tracks were then imported in Chemotaxis and Migration Tool (Ibidi) [28]. All the migration tracks were translated to a common starting point, (x, y) = (0,0) at t = 0. The migration speed was automatically generated along with the overlay image of all analyzed cell trajectories. Slow moving or nonmotile cells were excluded from the analysis by setting a threshold velocity of 0.01 µm/min or higher. Likewise, the cells that encountered neighboring cells during the migration were also excluded from the analysis. At least 60 cells were tracked per experiment with each experiment repeated independently at least two times. Further, to determine the biased cell movement along aligned collagen fibrils, an orientation parameter < cos^2^θ > was defined by measuring the time-averaged cosine of the angle between the cell displacement vector and the fibril alignment axis over the tracking period. A value closer to 1 indicates strong guidance along the aligned collagen fibrils, whereas a value of 0.5 indicates random migration, and 0 indicates alignment perpendicular to the aligned collagen. Unlike the speed and total path length that are dependent on the tracks being a 2D projection of 3D migration, and do not account for the impact of directional persistence on speed estimation [15, 29], the values of < cos^2^θ > are independent of migration out of the projection plane and accurately measure a contact guidance response.

### Statistical Analysis

The Shapiro–Wilk normality test was employed to test the distribution for normality. The difference between the control and treatment groups was tested using an unpaired t-test with Welch’s correction or non-parametric Mann-Whitney U test. The data was presented as mean ± standard deviation. A *P*-value < 0.05 has been considered statistically significant. The statistical analysis was performed using Graphpad Prism software version 9.3. The significance value and sample size used for analysis are detailed in the respective figure legends, with each data point in the graph representing an individual cell. All the experiments were repeated independently at least twice, and more than twice if the two experiments were not self-consistent. Further, to evaluate potential differences in treatment effects across independent experimental repeats, a multiple linear regression analysis was conducted. Data from three to four experimental repeats were combined into a single dataset for this analysis. A binary indicator variable was used to represent the treatment condition (e.g., 0 assigned to MOCK and 1 assigned to CSPG4^WT^). Additionally, a categorical variable was created to numerically code each experiment (from 1 to 4) to indicate the source of data from each independent repeat. To assess whether the treatment effect varied across experiments, interaction terms between the treatment and the experiment were included. These interaction terms were created by multiplying the treatment variable (0 or 1) by the experiment indicator (1 to 4). This approach allowed the model to examine differences in treatment effects across experiments. The regression analysis was then performed using Microsoft Excel Data Analysis Toolpak. A P-value below 0.05 for the treatment effect indicated that the treatment group had a significant impact on cell guidance and migration speed, regardless of variability between experiments. Any use of “difference” in describing results implies statistical significance as defined here.

## Results

**Fig. 1:**
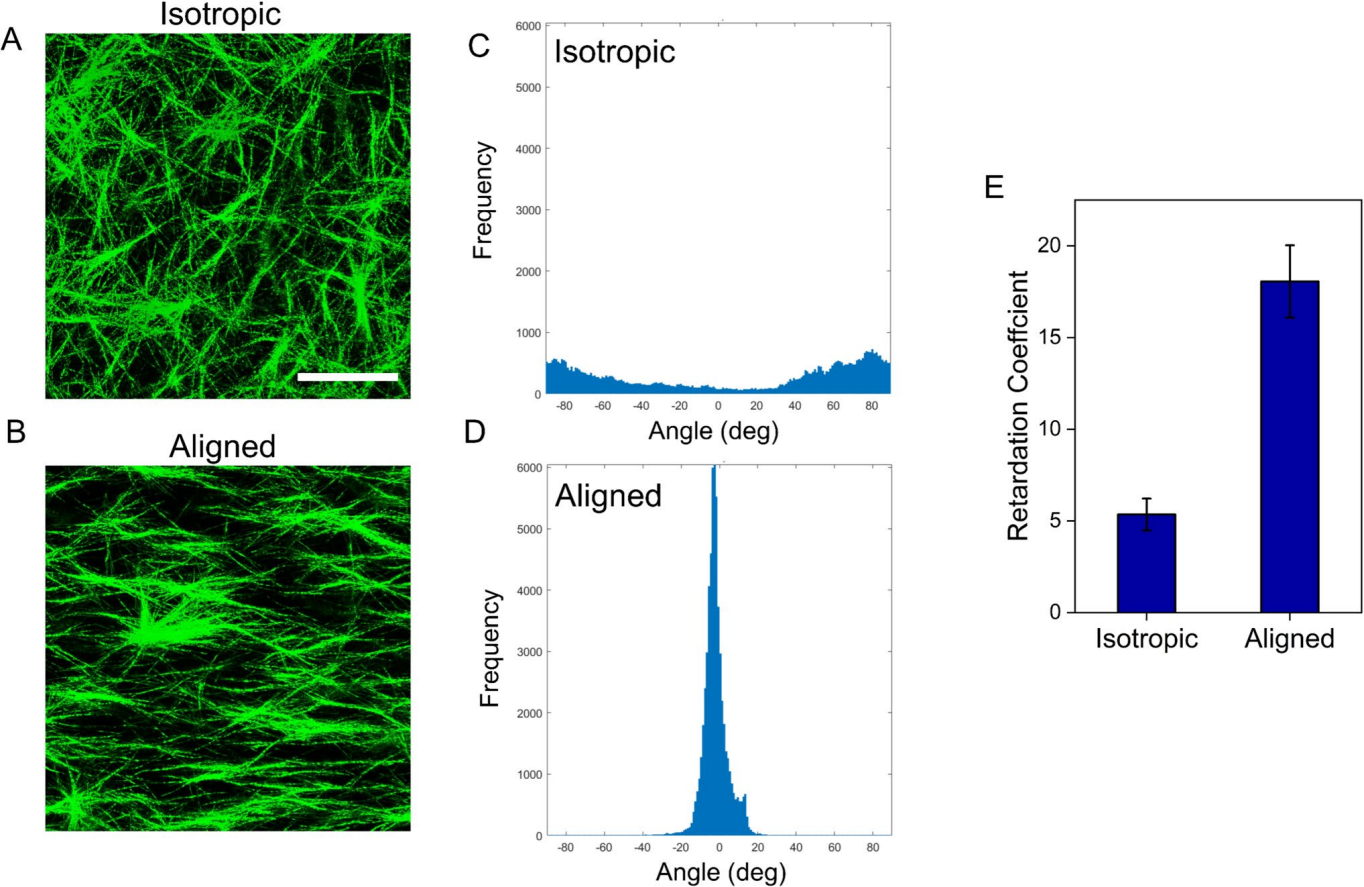
Magnetic alignment of collagen fibrils. (**A**, **B**) Comparison of confocal reflectance images showing the collagen fibril orientation in isotropic control vs. aligned collagen gels. Scale bar: 10 µm. (**C**, **D**, **E**) Assessment of the extent of fibril alignment by polarized light microscopy. Histogram showing the angular distribution of fiber alignment in isotropic (**C**) vs. aligned (**D**) collagen gels. (**E**) The gel retardation coefficient, a measure of fibril alignment strength, is markedly higher in the aligned collagen gels

To generate aligned 3D collagen tracks as guidance fields, the pre-gel collagen mixture containing cells was exposed to a 9.4 T magnetic field following neutralization to induce fibril orientation during collagen self-assembly. The negative diamagnetic anisotropy of collagen enables permanent fibril alignment in the direction perpendicular to the applied magnetic field when fibrillogenesis (gel formation) occurs in a sufficiently small gap [30]. A transparent U-shaped cell culture chamber with a high aspect ratio was employed to enable uniform magnetic alignment and continuous light microscopic visualization of cell migration. Fig. 1A, B show the collagen microstructure and fibril alignment in the absence or presence of a magnetic field, respectively. A relatively random angular distribution of fibrils (Fig. 1C) indicates no preferred orientation in the isotropic gel (without magnetic field exposure), whereas most of the fibrils were oriented within ± 10° in gels exposed to high strength magnetic field (Fig. 1D). Quantification of the gel retardation coefficient (Fig. 1E) further confirms greater birefringence exhibited by collagen gels magnetically aligned in a 9.4 T magnetic field.

### Contact Guidance of Melanoma Cells *in Vitro* is Enhanced by CSPG4 Expression

**Fig. 2:**
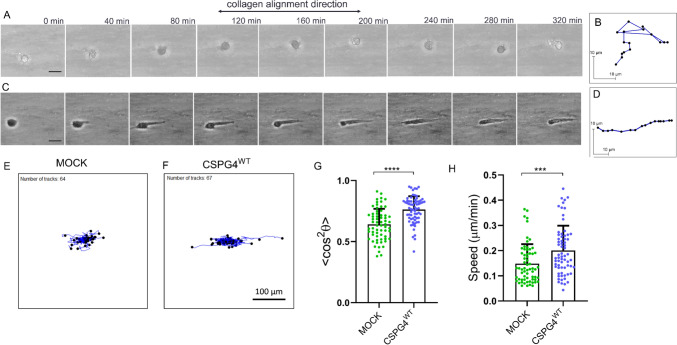
CSPG4 facilitates guidance sensing of melanoma cells in aligned collagen gel. WM1552C melanoma cells differentially expressing CSPG4 were entrapped in magnetically aligned collagen gel, and cell migration was monitored by time-lapse video microscopy using the EVOS microscope (original magnification × 20). **A**, **C** Time-lapse sequences of representative cells: Cell migration was largely random for the MOCK cells (**A**), whereas the CSPG4^WT^ cell exhibited persistent movement along the direction of collagen alignment (**C**). Scale bar: 5 µm. Cell tracks of the MOCK and CSPG4^WT^ cells in (**A**) and (**C**) are presented in (**B**) and (**D**), respectively. The trajectory is shown in blue with a total length of 6 h with each point 20 min apart. **E**, **F** Cell tracks for a population (*n* > 60) of MOCK and CSPG4^WT^ cells in collagen gels aligned in the horizontal direction. Initial cell positions were translated to the origin. Each blue line represents an individual cell track, and the solid black circle indicates its endpoints. **G** Cell orientation parameter < cos^2^θ > derived from the tracks is greater for CSPG4^WT^ cells compared to MOCK cells (0 = perfect vertical, 0.5 = random, 1 = perfect horizontal orientation). **H** Cell speed is also greater for CSPG4^WT^ cells compared to MOCK cells. Data presented as Mean ± SD. *n* = 64 and 67 cells for MOCK and CSPG4 cells, respectively. One of 3 independent experiments is shown here (Expt #1). Two-tailed Mann-Whitney test, ****P ≤ 0.0001, ***P ≤ 0.001

Next, to investigate the specific role of CSPG4 in mediating melanoma contact guidance, WM1552 cells were stably transfected to express the proteoglycan by transfection with vector (pcDNA 3.1(+)) containing full-length CSPG4. The same cells transfected with mock plasmid served as the control. Both mock-transfected (“MOCK”) and CSPG4 expressing (“CSPG4^WT^”) cells were entrapped in 9.4 T magnetically-aligned collagen gels, and the guidance response was compared by tracking cell positions over 6-7 hr duration (Fig. 2A–H). The representative time-lapse sequence of a single CSPG4^WT^ cell in Fig. 2A–D reveals biased migration along the axis of collagen fibril alignment, as opposed to the random motility of MOCK cells that endogenously lacked CSPG4. Trajectories of multiple cells (> 60) translated to a common origin indicate greater guidance response by CSPG4^WT^ cells compared with MOCK cells, exhibiting little or no apparent directionality (Fig. 2E,F). Corresponding cell trajectory analysis further confirms the greater guidance response by CSPG4^WT^ cells with〈cos^2^θ〉closer to the “perfect” guidance value of 1 compared with MOCK cells with〈cos^2^θ〉closer to the random motility value of 0.5 (****P ≤ 0.0001, Fig. 2G). Likewise, the migration speed was found to be greater for the CSPG4^WT^ cells (***p ≤ 0.001; Fig. 2H). Results across all 3 independent experiments showed consistent differences (Supplementary Table 1). Altogether, these results indicate an increased contact guidance sensitivity of the CSPG4 expressing cells (“CSPG4^WT^”).

### Deletion of the Cytoplasmic Domain in CSPG4 Decreased Contact Guidance, But Deletion of Only the PDZ Binding Domain Increased Contact Guidance

**Fig. 3:**
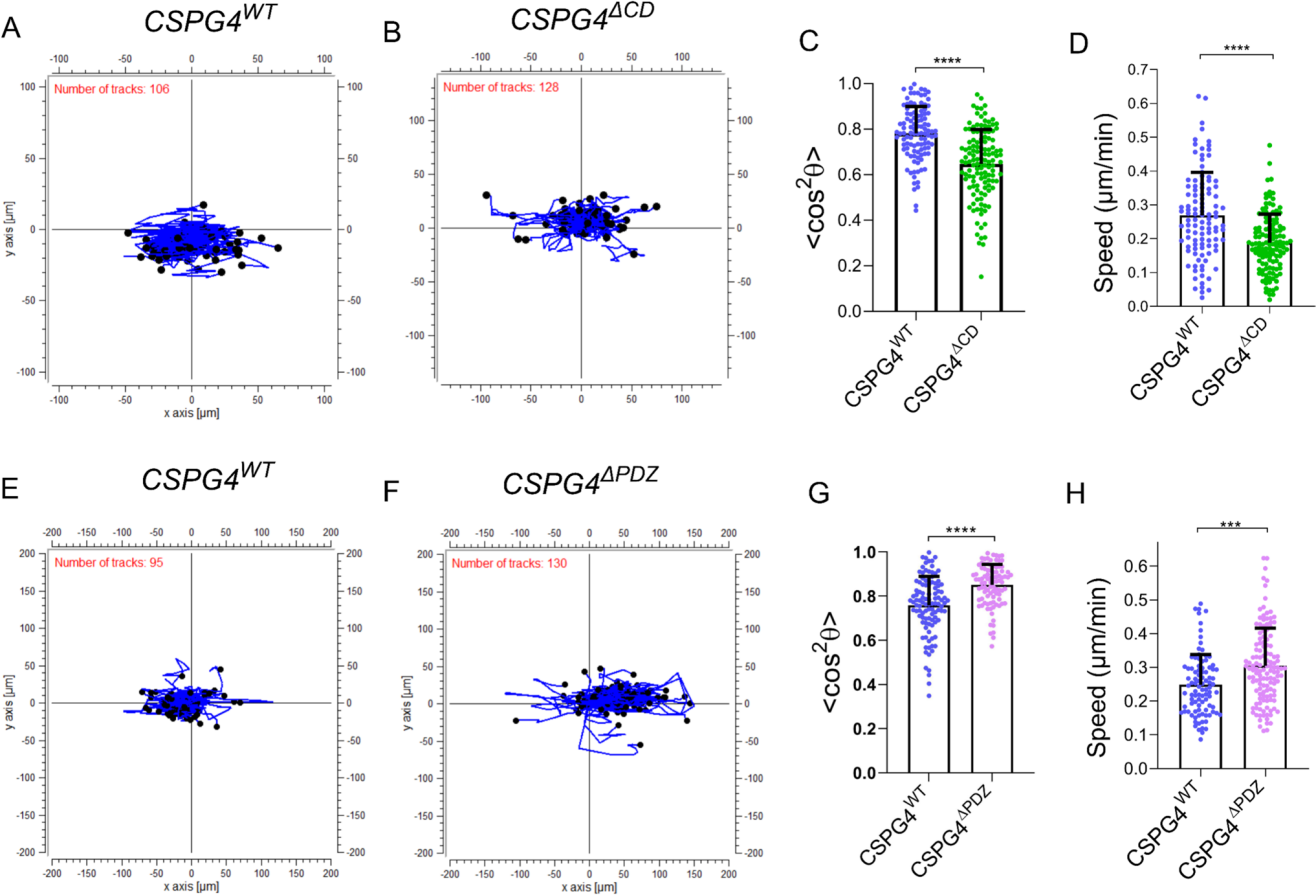
Contact guidance in aligned collagen gel was reduced with deletion of the cytoplasmic domain of CSPG4, but increased with deletion of the intracellular PDZ binding motif. (**A**–**D**) Effect of cytoplasmic domain deletion. (**A**, **B**) Overlay of cell tracks in aligned collagen gels as in Fig. 1 for CSPG4^WT^ cells and CSPG4ΔCD cells. (**C**, **D**) Deletion of the entire cytoplasmic region of CSPG4 reduced the contact guidance and cell speed of the mutant cells (CSPG4ΔCD) compared to the CSPG4 over-expressing cells (CSPG4^WT^). n = 106 cells for CSPG4^WT^ and 128 cells for CSPG4^ΔCD^. Data representative of 2 independent experiments. ****P ≤ 0.0001; Unpaired t-test with Welch’s correction. (**E**–**H**) Effect of PDZ binding domain deletion. (**E**, **F**) Overlay of cell tracks in aligned collagen gels for cells transfected with CSPG4 having deletion of the PDZ binding domain (CSPG4^ΔPDZ^) in comparison to CSPG4WT cells. (**G**) Plot of cell orientation parameter < cos^2^θ > and (**H**) migration speed showing increases for CSPG4^ΔPDZ^ cells in comparison to CSPG4^WT^ cells in this experiment. For < cos^2^θ > , *n* = 104 cells for CSPG4^WT^ and 93 for CSPG4^ΔPDZ^ cells. ****P ≤ 0.0001. For speed measurements, n = 95 and 129 cells, respectively; analyzed by Mann Whitney U-test. Data presented as Mean ± SD. 1 of 4 independent experiments is shown here (Expt #4). See text for statistical analysis of the pooled data from all 4 experiments

Having identified the critical involvement of CSPG4 in melanoma contact guidance, we next explored the candidate molecular mechanisms linking CSPG4-mediated guidance to intracellular signaling cascades. As a first step, we stably transfected and expressed CSPG4 lacking a cytoplasmic domain (CSPG4^ΔCD^) to investigate the function of this domain in sensing the aligned collagen fibrils. As depicted in Fig. 3A–D, the ΔCD mutant exhibited substantially diminished contact guidance measured as reduced < cos^2^θ > , and reduced migration speed, indicating the importance of the CSPG4 cytoplasmic domain that contains multiple sites potentially crucial for downstream signaling. One is the PDZ binding domain implicated in the binding of CSPG4 to PDZ-containing scaffold proteins (e.g. syntenin) and subsequent activation of the focal adhesion kinase (FAK) pathway. Hence, to interrogate the possible role of PDZ in contact guidance signaling, we transfected cells to express CSPG4 lacking the PDZ binding domain (CSPG4^ΔPDZ^ mutant) and compared their contact guidance to CSPG4^WT^ cells (Fig. 3E, F). Counterintuitively, there was an increase in the contact guidance sensitivity upon removal of the PDZ binding domain as shown in Fig. 3G**.** There was also an increase in the migration speed (Fig. 3H) in the experiment summarized in Fig. 3. Results were not consistent across all 4 independent experiments for this group. A linear mixed model analysis, which accounts for experimental variability, performed on the pooled data from the 4 experiments confirmed an increase of contact guidance (*P* < 0.001) for the CSPG4^ΔPDZ^ cells compared to CSPG4^WT^ cells (Supplementary Table 1). In contrast, the migration speed was not different (*P* = 0.31).

### Phosphoacceptor site (Thr^2310^) in the Cytoplasmic Domain of CSPG4 is Required for Melanoma Contact Guidance

**Fig. 4:**
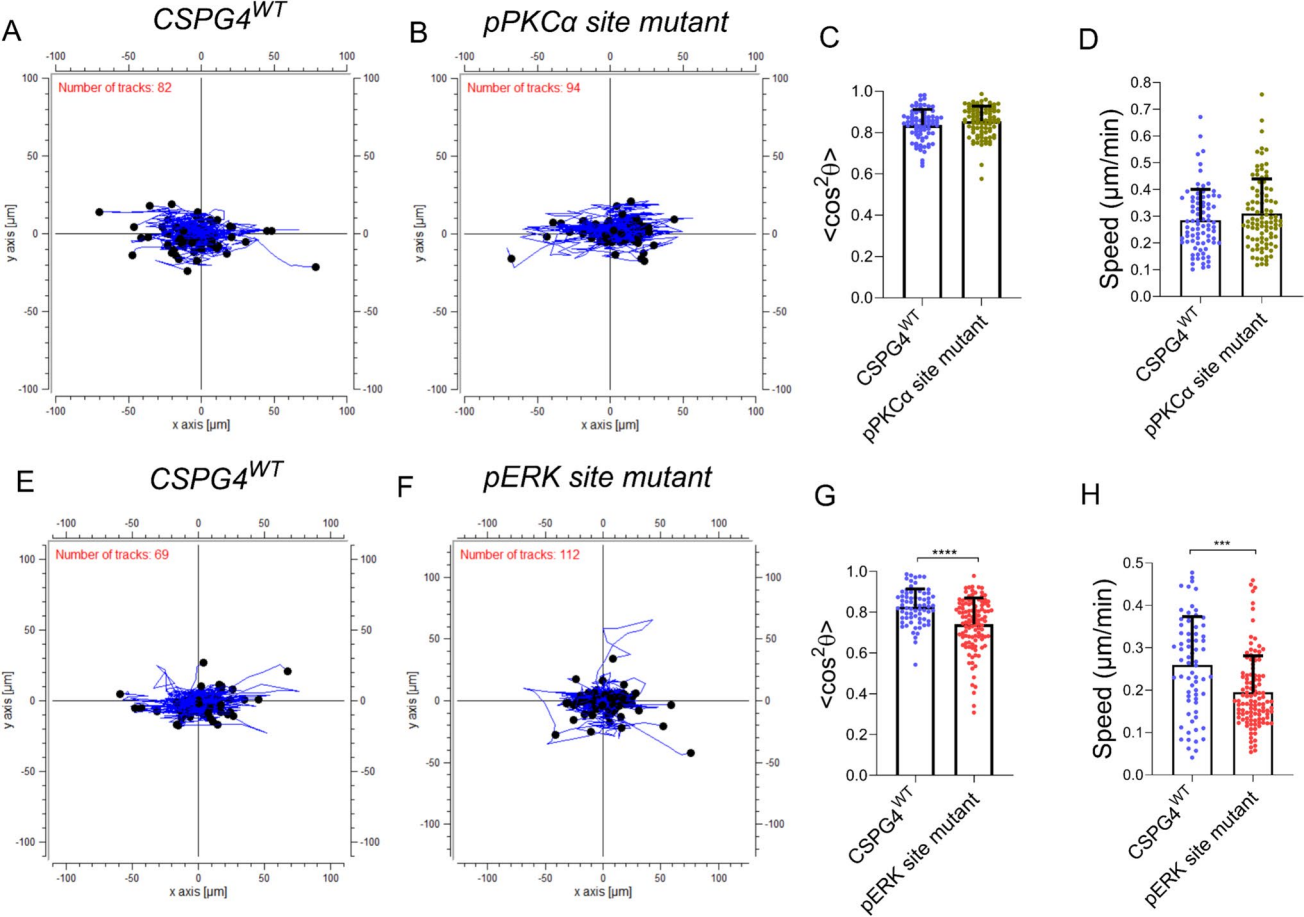
pERK-mediated phosphorylation of CSPG4 is critical for contact guidance of melanoma cells in aligned collagen gel. **A**–**D** Comparison of guided migration in WM1552C cells transfected with wild-type or pPKCα site mutant CSPG4. Overlay of displacement tracks (blue solid line) for all analyzed cells in CSPG^WT^ and pPKCα site mutant groups are shown in **A** and **B**. Respective quantitation of oriented migration (**C**) and speed **D** indicates no impact of PKCα phospho-mutant on the contact guidance sensing ability. *n* = 82 cells for CSPG4^WT^; *n* = 94 cells for pPKCα site mutant; Mann Whitney U test showed no difference between the two groups. **E**–**H** Point mutation at the ERK phosphorylation site (Thr2310) diminished the guidance sensing ability of CSPG4-expressing melanoma cells. Plot of **G** cell orientation parameter < cos^2^θ > and **H** migration speed showing a decrease for the ERK phospho-mutant cells in comparison to CSPG4^WT^ cells in this experiment. *n* = 69 cells for CSPG4^WT^; *n* = 112 cells for pERK site mutant. Welch’s t-test, ***P ≤ 0.01, ****P ≤ 0.0001. Data presented as Mean ± SD. Results from 1 of 2 independent experiments are shown here (Expt #2). pPKCα site mutant – WM1552C cells expressing CSPG4 mutated at Thr2252 site to valine, pERK site mutant – WM1552C cells expressing CSPG4 mutated at site Thr2310 to valine

In addition to the PDZ binding motif, the intracellular domain of CSPG4 has two prominent phosphoacceptor sites, namely Thr^2252^, phosphorylated by pPKCα and Thr^2310^, phosphorylated by pERK 1,2 respectively. These sites are shared with the rat homologue NG2, and previous studies have implicated both of these phosphoacceptor sites in NG2 stimulated motility and growth [25]. We therefore mutated these two phosphoacceptor threonine to valine to render them inactive to examine their potential impact in CSPG4-mediated contact guidance. Cells expressing the CSPG4 pPKCα phosphoacceptor site mutant did not exhibit any measurable difference in contact guidance or cell speed compared to that observed in cells expressing the CSPG4^WT^ construct Fig. 4A-D. In striking contrast, expression of the CSPG4 pERK 1,2 phosphoacceptor site mutant caused cells to exhibit both reduced contact guidance (Fig. 4E-G) and cell speed (Fig. 4H). These results were consistent between 2 independent experiments for this group, with the differences from the CSPG4^WT^ cells confirmed in the linear mixed model analysis (Supplementary Table 1).

**Fig. 5:**
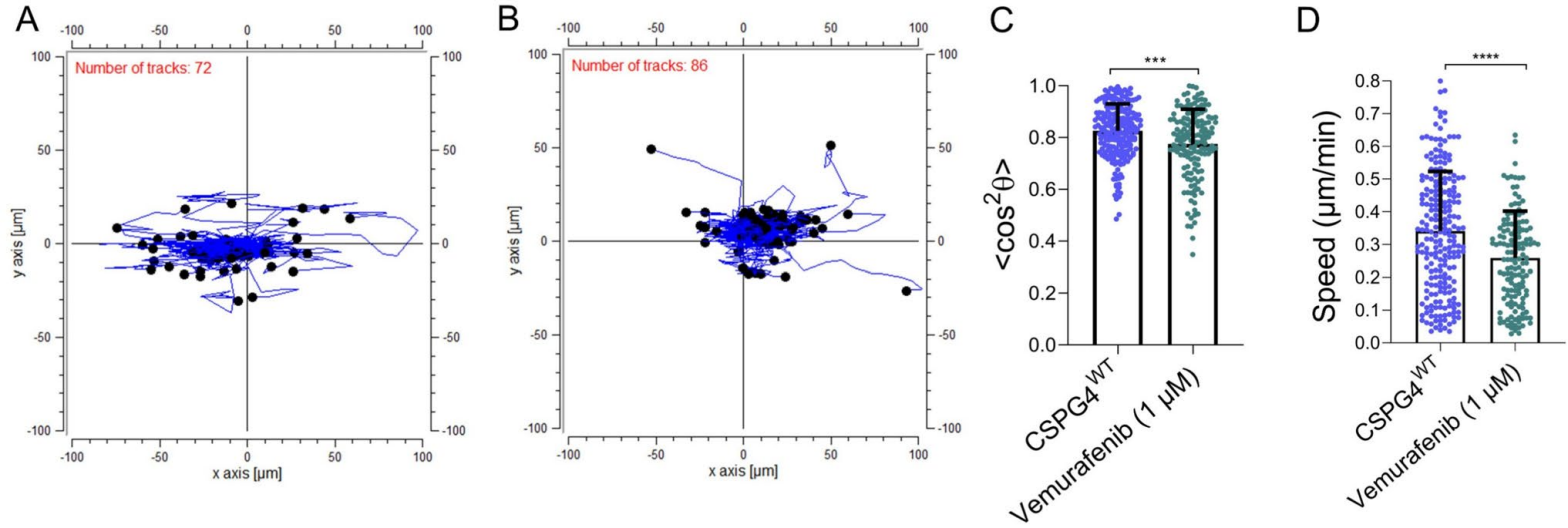
Inhibitor of mutant active BRAF causes reduced CSPG4-mediated contact guidance sensing in aligned collagen gel**s**. CSPG4^WT^ cells were incubated overnight in the presence or absence of the mutant active BRAF inhibitor vemurafenib prior to trypsinization and entrapment within aligned collagen gel. Time-lapse images were captured every 20 min to determine the effect of the pharmacological treatment on the cell migration. **A**, **B** Comparison of cell tracks for untreated (**A**) vs vemurafenib treated (**B**) CSPG4^WT^ cells, indicating a reduction in contact guidance, confirmed by measurement of the orientation parameter < cos^2^θ > (**C**), as well as reduced migration speed (**D**) Results from 1 of 2 independent experiments are shown here (Expt #2). Bars represent mean ± SD. *n* = 225 cell tracks for untreated CSPG^WT^ controls, *n* = 146 for Vemurafenib (1 µM) treated cells. ***P ≤ 0.001, ****P ≤ 0.0001, Mann Whitney U Test

We also performed pharmacological experiments to inhibit mutant active V600E BRAF, which these cells express and which requires CSPG for activation [10]. CSPG4^WT^ cells were preincubated overnight with 1 μM of the mutant active BRAF inhibitor vemurafenib, prior to entrapment within aligned collagen gel. As shown in Fig. 5A-D, treatment with vemurafenib resulted in a reduction in contact guidance and migration speed compared to untreated control cells. These results were consistent between 2 independent experiments for this group, with the differences from the untreated CSPG4^WT^ cells confirmed in the linear mixed model analysis (Supplementary Table 1).

## Discussion and Conclusion

CSPG4 expression in cutaneous melanoma cells is well documented [1, 2, 11]. Recent immunohistochemical analysis of 189 melanoma tissue samples demonstrated that almost 2/3 (65.6%) contained CSPG4-positive tumor cell subsets. Subtype comparisons indicated that CSPG4 staining was higher in superficial spreading melanomas [11]. CSPG4 expression was detected in both primary and metastatic melanomas, further supporting its investigation as a viable therapeutic target. These results in patients are consistent with other studies showing that CSPG4 expression is detected and functional in melanoma cells isolated from both primary and metastatic lesions [1, 6, 7, 24, 31].

At the cellular level, CSPG4 functions as a plasma membrane associated signal transduction node to enhance melanoma progression. Numerous studies have demonstrated the complexities of multiple structural/functional domains of CSPG4 that can enhance tumor cell adhesion, motility, mesenchymal transition, invasion and growth [32, 33]. For example, CSPG4 stimulates integrin function by activating c-src to promote focal adhesion kinase activation and integrin-mediated cell adhesion, cytoskeletal reorganization/cell spreading, and motility [7, 24, 31]. CSPG4 expression has been functionally linked to enhanced cell growth by promoting activation of the ERK 1,2 pathway via its impact on mutant active BRAF function, expressed in the WM15552 cells used in the current study [7]. CSPG4 has been immunolocalized to filopodia [34] and it promotes activation/phosphorylation of p130cas by a cdc42-associated non-membrane tyrosine kinase (Ack1) [35], linking it to actin polymerization, filopodial extension and/or function. Thus, CSPG4 functions to promote complex and dynamic changes in both membrane-associated lamellipodia and filopodia, both of which are important for mediating contact guidance [36, 37].

The current study demonstrates that CSPG4 function can also stimulate both contact guidance and migration speed in cells presented with an aligned collagen fibril matrix, as would be encountered by locally invasive radial growth phase tumor cells in the immediate vicinity of primary melanoma lesions. While these studies were not designed to specifically define which of the several reported CSPG4 functions are responsible for increased migration speed and contact guidance, the results have implications for clinical staging and therapy in patients harboring primary melanomas. Cell track metrics analyzed during a 6-8 hour period of migration within gels of magnetically-aligned collagen fibrils demonstrated that CSPG4 expression promoted both directional bias and an increase in cell speed of RGP melanoma cells relative to CSPG4 null counterparts. The studies also identified several key CSPG4 core protein structural/functional features important for contact guidance [7, 24, 31]. There was a reduction in contact guidance and speed for cells expressing a CSPG4 construct in which the cytoplasmic domain was truncated, consistent with a previous study [31], emphasizing the importance of key signaling domains in this region of the CSPG core protein in the current experimental model.

**Fig. 6:**
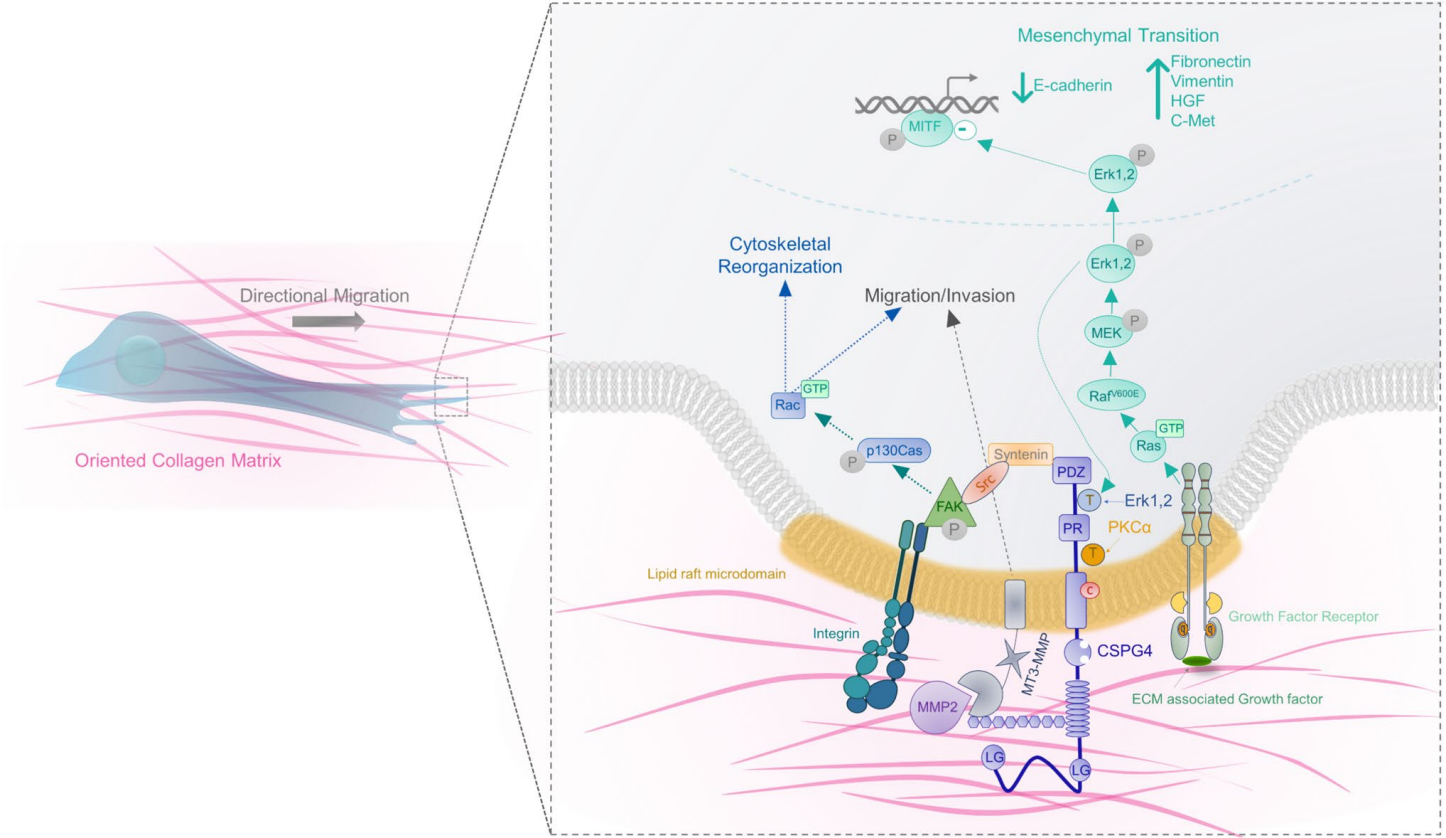
Chondroitin sulfate proteoglycan 4 (CSPG4) Signaling Pathways. CSPG4 functions to activate two major overlapping but distinct signaling cascades: integrin/focal adhesion kinase (FAK) signaling involving binding to syntenin via the PDZ motif binding domain, and MAPK pathway signaling leading to ERK1/2 activation. Through these two branches of cytoplasmic domain signaling, CSPG4 ultimately promotes tumor migration and invasion by modifying a variety of phenotypic functions, as shown here. The figure is modified and based on previous work [31]

As a result, we also evaluated CSPG4 constructs that had specific sites within the cytoplasmic domain mutated. These sites included either of two key phosphoacceptor sites (pERK 1,2 or PKAα) or the carboxyl terminal PDZ binding motif. Site specific mutation of the pERK 1,2 phosphoacceptor site resulted in decreased contact guidance and migration speed, whereas there was no detectable impact of mutating the PKCα phosphoacceptor site. This leads us to conclude that a phosphorylated ERK 1,2 phosphoacceptor site within the cytoplasmic domain is implicated in the CSPG4-stimulated cell motility (Fig. 6) [7, 9, 31, 38]. By contrast, deletion of the carboxyl-terminal PDZ motif binding domain produced results that were distinct from those observed using phospho-acceptor site mutants. Although we have previously shown that the CSPG4 PDZ domain binding motif binds syntenin [31, 38] it needs to be emphasized that there are additional intracellular PDZ motif containing proteins (e.g. MUPP1 or GRIP1) both of which impact signaling pathways independent of syntenin [1]. Thus, although the current results identify key structural domains important for CSPG4-enhanced motility, a mechanistic understanding of these results will require studies in which these mutated constructs are integrated with experiments that interrogate key oncogenic signaling pathways components.

Melanoma therapy has undergone significant and rapid improvements over the last two decades [39]. Importantly, we also demonstrate that vemurafenib, a clinically used mutant active BRAF inhibitor, inhibits CSPG4 stimulated motility and contact guidance. While inhibiting mutant active BRAF is well known for inhibiting melanoma tumor growth in patients, the current studies indicate that this inhibitor could also function to limit localized tumor cell invasion into the oriented collagen-rich microenvironment of primary tumors. This result has novel mechanistic implications for vemurafenib to limit radial to vertical growth phase progression in primary tumors. Furthermore, while immune-based strategies dominate the therapeutic landscape, there is still a role for small- molecule inhibitors such as vemurafenib combined with immune checkpoint inhibitors [39]. CSPG4 is widely considered as a viable immune target in multiple tumors, including melanoma [1]. Recent clinical studies indicate that immunotherapy combined with small-molecule therapy may be superior to therapies focused on one or the other approach [40]. Since the current results indicate that CSPG4 expression would give melanoma cells a selective advantage in malignant invasion, it is reasonable to speculate that CSPG4-expressing melanoma cells would be particularly vulnerable to this combination therapy.

Limitations of this study, besides those discussed above regarding elucidation of signaling pathways, include omission of MOCK transfection control samples in the experiments except for the experiments to establish the effect of CSPG4 expression (Fig. 2). This was due to technical limitations associated with live cell imaging. Thus, we cannot conclude the observed reductions in speed and guidance in treatments are complete or partial relative to the MOCK level that would have been measured, only that the treatment is implicated.

## Supplementary Information

Below is the link to the electronic supplementary material.

Supplementary file1 (DOCX 17 KB)

## Data Availability

The data supporting the findings of this study are available within the manuscript and its supplementary materials.

## References

[1] Price, M. A., L. E. Colvin Wanshura, J. Yang, J. Carlson, B. Xiang, G. Li, S. Ferrone, A. Z. Dudek, E. A. Turley, and J. B. McCarthy. CSPG4, a potential therapeutic target, facilitates malignant progression of melanoma. *Pigment cell melanoma res.* 24(6):1148–1157, 2011. 22004131 10.1111/j.1755-148X.2011.00929.x PMC3426219

[2] Nicolosi, P. A., A. Dallatomasina, and R. Perris. Theranostic impact of NG2/CSPG4 proteoglycan in cancer. *Theranostics*. 5(5):530–544, 2015. 25767619 10.7150/thno.10824 PMC4350014

[3] Inamdar, G. S., S. V. Madhunapantula, and G. P. Robertson. Targeting the MAPK pathway in melanoma: why some approaches succeed and other fail. *Biochem. Pharmacol.* 80(5):624–637, 2010. 20450891 10.1016/j.bcp.2010.04.029 PMC2897908

[4] Orgaz, J. L., and V. Sanz-Moreno. Emerging molecular targets in melanoma invasion and metastasis. *Pigment cell melanoma res.* 26(1):39–57, 2013. 23095214 10.1111/pcmr.12041

[5] Lemech, C., J. Infante, and H. T. Arkenau. The potential for BRAF V600 inhibitors in advanced cutaneous melanoma: rationale and latest evidence. *Therapeutic adv. Med. oncology*. 4(2):61–73, 2012. 10.1177/1758834011432949 PMC3296083 22423265

[6] Uranowska, K., T. Kalic, V. Valtsanidis, M. Kitzwögerer, H. Breiteneder, and C. Hafner. Expression of chondroitin sulfate proteoglycan 4 (CSPG4) in melanoma cells is downregulated upon inhibition of BRAF. *Oncology reports.* 45(4):14, 2021. 33649790 10.3892/or.2021.7965 PMC7876987

[7] Yang, J., M. A. Price, G. Y. Li, M. Bar-Eli, R. Salgia, R. Jagedeeswaran, J. H. Carlson, S. Ferrone, E. A. Turley, and J. B. McCarthy. Melanoma proteoglycan modifies gene expression to stimulate tumor cell motility, growth, and epithelial-to-mesenchymal transition. *Cancer Res.* 69(19):7538–7547, 2009. 19738072 10.1158/0008-5472.CAN-08-4626 PMC2762355

[8] Zengarini, C., M. Mussi, G. Veronesi, A. Alessandrini, M. Lambertini, and E. Dika. BRAF V600K vs. BRAF V600E: a comparison of clinical and dermoscopic characteristics and response to immunotherapies and targeted therapies. *Clinical exp. dermatol.* 47(6):1131–1136, 2022. 10.1111/ced.15113 PMC9311196 35080260

[9] Lu, H., S. Liu, G. Zhang, L. N. Kwong, Y. Zhu, J. P. Miller, Y. Hu, W. Zhong, J. Zeng, L. Wu, C. Krepler, K. Sproesser, M. Xiao, W. Xu, G. C. Karakousis, L. M. Schuchter, J. Field, P. J. Zhang, M. Herlyn, X. Xu, and W. Guo. Oncogenic BRAF-Mediated Melanoma Cell Invasion. *Cell Reports*. 15(9):2012–2024, 2016. 27210749 10.1016/j.celrep.2016.04.073 PMC4889462

[10] Kim, A., and M. S. Cohen. The discovery of vemurafenib for the treatment of BRAF-mutated metastatic melanoma. *Expert opinion on drug discovery*. 11(9):907–916, 2016. 27327499 10.1080/17460441.2016.1201057 PMC5443413

[11] Grossauer, A., K. Uranowska, M. Kitzwögerer, M. Mostegel, H. Breiteneder, and C. Hafner. Immunohistochemical detection of the chondroitin sulfate proteoglycan 4 protein in primary and metastatic melanoma. *Oncology lett.* 26(3):382, 2023. 10.3892/ol.2023.13968 PMC10407859 37559576

[12] van Kempen, L. C., J. Rijntjes, I. Mamor-Cornelissen, S. Vincent-Naulleau, M. J. Gerritsen, D. J. Ruiter, M. C. van Dijk, C. Geffrotin, and G. N. van Muijen. Type I collagen expression contributes to angiogenesis and the development of deeply invasive cutaneous melanoma. *Intern. J. cancer*. 122(5):1019–1029, 2008. 10.1002/ijc.23147 17957794

[13] Ray, A., and P. P. Provenzano. Aligned forces: Origins and mechanisms of cancer dissemination guided by extracellular matrix architecture. *Curr. opinion cell biol*. 72:63–71, 2021. 34186415 10.1016/j.ceb.2021.05.004 PMC8530881

[14] Provenzano, P. P., K. W. Eliceiri, D. R. Inman, and P. J. Keely. Engineering three-dimensional collagen matrices to provide contact guidance during 3D cell migration. *Curr. protoc. cell boil. Chapter 10.* 47(1):10–17, 2010. 10.1002/0471143030.cb1017s47 PMC3905986 20521230

[15] Dickinson, R. B., S. Guido, and R. T. Tranquillo. Biased cell migration of fibroblasts exhibiting contact guidance in oriented collagen gels. *Annals biomed. eng.* 22(4):342–356, 1994. 10.1007/BF02368241 7998680

[16] Conklin, M. W., J. C. Eickhoff, K. M. Riching, C. A. Pehlke, K. W. Eliceiri, P. P. Provenzano, A. Friedl, and P. J. Keely. Aligned collagen is a prognostic signature for survival in human breast carcinoma. *The Am. J. pathol*. 178(3):1221–1232, 2011. 21356373 10.1016/j.ajpath.2010.11.076 PMC3070581

[17] Beeghly, G. F., K. Y. Amofa, C. Fischbach, and S. Kumar. Regulation of tumor invasion by the physical microenvironment: lessons from breast and brain cancer. *Annu. Rev. biomed. Eng.* 24:29–59, 2022. 35119915 10.1146/annurev-bioeng-110220-115419 PMC9177572

[18] Provenzano, P. P., K. W. Eliceiri, J. M. Campbell, D. R. Inman, J. G. White, and P. J. Keely. Collagen reorganization at the tumor-stromal interface facilitates local invasion. *BMC Medicine*. 4(1):38, 2006. 17190588 10.1186/1741-7015-4-38 PMC1781458

[19] Weigelin, B., G. J. Bakker, and P. Friedl. Intravital third harmonic generation microscopy of collective melanoma cell invasion: Principles of interface guidance and microvesicle dynamics. *Intravital*. 1(1):32–43, 2012. 29607252 10.4161/intv.21223 PMC5858865

[20] Wang, J., J. W. Petefish, A. C. Hillier, and I. C. Schneider. Epitaxially Grown Collagen Fibrils Reveal Diversity in Contact Guidance Behavior among Cancer Cells. *Langmuir*. 31(1):307–314, 2015. 25531276 10.1021/la503254x PMC4295811

[21] Nuhn, J. A. M., A. M. Perez, and I. C. Schneider. Contact guidance diversity in rotationally aligned collagen matrices. *Acta. biomaterialia*. 66:248–257, 2018. 29196116 10.1016/j.actbio.2017.11.039 PMC5750117

[22] Fischer, R. S., X. Sun, M. A. Baird, M. J. Hourwitz, B. R. Seo, A. M. Pasapera, S. B. Mehta, W. Losert, C. Fischbach, J. T. Fourkas, and C. M. Waterman. Contractility, focal adhesion orientation, and stress fiber orientation drive cancer cell polarity and migration along wavy ECM substrates. *Proc. Natl. Acad. Sci. U.S.A.* 118(22):e201135118, 2021. 10.1073/pnas.2021135118 PMC8179222 34031242

[23] Guido, S., and R. T. Tranquillo. A methodology for the systematic and quantitative study of cell contact guidance in oriented collagen gels. Correlation of fibroblast orientation and gel birefringence. *J. Cell Sci.* 105:317–331, 1993. 8408268 10.1242/jcs.105.2.317

[24] Yang, J., M. A. Price, C. L. Neudauer, C. Wilson, S. Ferrone, H. Xia, J. Iida, M. A. Simpson, and J. B. McCarthy. Melanoma chondroitin sulfate proteoglycan enhances FAK and ERK activation by distinct mechanisms. *The J. cell boil.* 165(6):881–891, 2004. 10.1083/jcb.200403174 PMC2172406 15210734

[25] Makagiansar, I. T., S. Williams, T. Mustelin, and W. B. Stallcup. Differential phosphorylation of NG2 proteoglycan by ERK and PKCalpha helps balance cell proliferation and migration. *The J. cell boil.* 178(1):155–165, 2007. 10.1083/jcb.200612084 PMC2064431 17591920

[26] Tower, T. T., M. R. Neidert, and R. T. Tranquillo. Fiber alignment imaging during mechanical testing of soft tissues. *Annals Biomed. Eng.* 30(10):1221–1233, 2002. 10.1114/1.1527047 12540198

[27] C.-S. Jhun, M.C. Evans, V.H. Barocas, R.T. Tranquillo, (2009). Planar biaxial mechanical behavior of bioartificial tissues possessing prescribed fiber alignment, J. Biomech. Eng. 131(8) 10.1115/1.3148194 PMC3717317 19604018

[28] Zengel, P., A. Nguyen-Hoang, C. Schildhammer, R. Zantl, V. Kahl, and E. Horn. μ-Slide chemotaxis: a new chamber for long-term chemotaxis studies. *BMC Cell Biol.* 12(1):21, 2011. 21592329 10.1186/1471-2121-12-21 PMC3118187

[29] Dickinson, R. B., and R. T. Tranquillo. Optimal estimation of cell movement indices from the statistical analysis of cell tracking data. *AIChE J*. 39(12):1995–2010, 1993.

[30] Torbet, J., and M. C. Ronzière. Magnetic alignment of collagen during self-assembly. *Biochem J*. 219(3):1057–1059, 1984. 6743242 10.1042/bj2191057 PMC1153582

[31] Yang, J., M. A. Price, L. E. C. Wanshura, J. He, M. Yi, D. R. Welch, G. Li, S. Conner, J. Sachs, E. A. Turley, and J. B. McCarthy. Chondroitin sulfate proteoglycan 4 enhanced melanoma motility and growth requires a cysteine in the core protein transmembrane domain. *Melanoma res.* 29(4):365–375, 2019. 31140988 10.1097/CMR.0000000000000574 PMC6597303

[32] Innocenti, M. New insights into the formation and the function of lamellipodia and ruffles in mesenchymal cell migration. *Cell adhesion migr.* 12(5):401–416, 2018. 10.1080/19336918.2018.1448352 PMC6363039 29513145

[33] Iida, J., D. Pei, T. Kang, M. A. Simpson, M. Herlyn, L. T. Furcht, and J. B. McCarthy. Melanoma chondroitin sulfate proteoglycan regulates matrix metalloproteinase-dependent human melanoma invasion into type I collagen. *The J. boil. Chem.* 276(22):18786–18794, 2001. 10.1074/jbc.M010053200 11278606

[34] Garrigues, H. J., M. W. Lark, S. Lara, I. Hellström, K. E. Hellström, and T. N. Wight. The melanoma proteoglycan: restricted expression on microspikes, a specific microdomain of the cell surface. *The J. cell boil.* 103(5):1699–1710, 1986. 10.1083/jcb.103.5.1699 PMC2114375 2430975

[35] Eisenmann, K. M., J. B. McCarthy, M. A. Simpson, P. J. Keely, J. L. Guan, K. Tachibana, L. Lim, E. Manser, L. T. Furcht, and J. Iida. Melanoma chondroitin sulphate proteoglycan regulates cell spreading through Cdc42, Ack-1 and p130cas. *Nat. cell boil.* 1(8):507–513, 1999. 10.1038/70302 10587647

[36] Ramirez-San Juan, G. R., P. W. Oakes, and M. L. Gardel. Contact guidance requires spatial control of leading-edge protrusion. *Mol. boil. cell*. 28(8):1043–1053, 2017. 10.1091/mbc.E16-11-0769 PMC5391181 28228548

[37] Kubow, K. E., V. D. Shuklis, D. J. Sales, and A. R. Horwitz. Contact guidance persists under myosin inhibition due to the local alignment of adhesions and individual protrusions. *Sci. Rep.* 7(1):14380, 2017. 29085052 10.1038/s41598-017-14745-7 PMC5662575

[38] Macaulay, A. R. K., J. Yang, M. A. Price, C. L. Forster, M. J. Riddle, C. L. Ebens, F. W. Albert, A. Giubellino, J. B. McCarthy, and J. Tolar. Chondroitin sulfate proteoglycan 4 (CSPG4) increases invasion of recessive dystrophic epidermolysis bullosa-associated cutaneous squamous cell carcinoma by modifying TGFβ signaling. *The British j. dermatol.* 192(1):104–117, 2024. 10.1093/bjd/ljae295 PMC11663483 39018437

[39] Kennedy, L. B., and A. K. S. Salama. Multiple options: how to choose therapy in frontline metastatic melanoma. *Curr. Oncol. Rep.* 26(8):915–923, 2024. 38837107 10.1007/s11912-024-01547-0

[40] Atkins, M. B., P. A. Ascierto, D. Feltquate, J. L. Gulley, D. B. Johnson, N. I. Khushalani, J. Sosman, T. A. Yap, H. Kluger, R. J. Sullivan, and H. Tawbi. Society for Immunotherapy of cancer (SITC) consensus definitions for resistance to combinations of immune checkpoint inhibitors with targeted therapies. *J. Immunother. Cancer*. 11(3):e005923, 2023. 36918225 10.1136/jitc-2022-005923 PMC10016252

