# Supplementary material for: Melanoma/CSPG4-Enhanced Collagen-Mediated Contact Guidance Requires Mutant Active BRAF and the CSPG4 Core Protein Cytoplasmic Domain
Source: Cell Mol Bioeng. 2025 Dec 23;19(1):61–72. doi: 10.1007/s12195-025-00882-x (PMC13031561; doi:10.1007/s12195-025-00882-x)
Supplement: Supplementary file 1 — Supplementary file1 (DOCX 17 KB) [file 12195_2025_882_MOESM1_ESM.docx]

**Supplementary Table 1:** Summary of Statistical Analysis. Sample size, n = Number of cells, ns indicates not statistically significant. Statistical analysis was performed on each independent experiment and on the combined pool of all independent experimental repeats using a linear regression model to account for experimental variability.

| Treatment  No. | **Orientation Parameter**  **<cos^2^θ>** | Independent Experiments | | | | | | | | Combined |
| --- | --- | --- | --- | --- | --- | --- | --- | --- | --- | --- |
|  |  | **Exp #1** | | Exp #2 | | Exp #3 | | Exp #4 | | Linear Regression Analysis |
| 1 | MOCK | n=64 | P<0.001 | n=55 | P<0.001 | n=76 | P<0.001 |  | | P<0.001 |
|  | CSPG4^WT^ | n=67 |  | n=50 |  | n=83 |  |  |  |  |
| 2 | CSPG4^WT^ | n=65 | P<0.001 | n=106 | P<0.001 | - | | - | | P<0.001 |
|  | CSPG4^ΔCD^ | n=70 |  | n=128 |  |  |  |  |  |  |
| 3 | CSPG4^WT^ | n=104 | P<0.001 | n=113 | P<0.05 | n=134 | P<0.001 | n=95 | ns  P=0.730 | P<0.001 |
|  | CSPG4^ΔPDZ^ | n=93 |  | n=114 |  | n=99 |  | n=129 |  |  |
| 4 | CSPG4^WT^ | n=72 | ns  P=0.0975 | n=82 | ns  P=0.0841 | - | | - | | ns  P=0.904 |
|  | pPKCα | n=93 |  | n=94 |  |  |  |  |  |  |
| 5 | CSPG4^WT^ | n=75 | P<0.0001 | n=69 | P<0.0001 | - | | - | | P<0.001 |
|  | pERK | n=131 |  | n=112 |  |  |  |  |  |  |
| 6 | CSPG4^WT^ | n=152 | P<0.05 | n=225 | P<0.0001 | - | | - | | P<0.01 |
|  | Vemurafenib | n=62 |  | n=146 |  |  |  |  |  |  |

| Treatment  No. | **Migration speed** | Independent Experiments | | | | | | | | Combined |
| --- | --- | --- | --- | --- | --- | --- | --- | --- | --- | --- |
|  |  | Exp #1 | | Exp #2 | | Exp #3 | | Exp #4 | | Linear Regression Analysis |
| 1 | MOCK | n=64 | P<0.001 | n=55 | P<0.001 | n=76 | P<0.001 |  | | P<0.001 |
|  | CSPG4^WT^ | n=67 |  | n=50 |  | n=83 |  |  |  |  |
| 2 | CSPG4^WT^ | n=65 | P<0.001 | n=106 | P<0.001 | - | | - | | P<0.001 |
|  | CSPG4^ΔCD^ | n=70 |  | n=128 |  |  |  |  |  |  |
| 3 | CSPG4^WT^ | n=104 | P<0.01 | n=113 | P<0.01 | n=134 | P<0.01 | n=95 | P<0.001 | ns  P=0.31141 |
|  | CSPG4^ΔPDZ^ | n=93 |  | n=114 |  | n=99 |  | n=129 |  |  |
| 4 | CSPG4^WT^ | n=72 | ns  P=0.3116 | n=82 | ns  P=0.1805 | - | | - | | ns  P=0.8894 |
|  | pPKCα | n=93 |  | n=94 |  |  |  |  |  |  |
| 5 | CSPG4^WT^ | n=75 | P<0.0001 | n=69 | P<0.0001 |  | |  | | P<0.001 |
|  | pERK | n=131 |  | n=112 |  |  |  |  |  |  |
| 6 | CSPG4^WT^ | n=152 | ns  P=0.9275 | n=225 | P<0.0001 | - | | - | | P<0.01 |
|  | Vemurafenib | n=62 |  | n=146 |  |  |  |  |  |  |
